# Estimating presymptomatic episodic memory impairment using simple hand movement tests: A cross‐sectional study of a large sample of older adults

**DOI:** 10.1002/alz.13401

**Published:** 2023-07-30

**Authors:** Xinyi Wang, Rebecca J. St George, Aidan D. Bindoff, Alastair J. Noyce, Katherine Lawler, Eddy Roccati, Larissa Bartlett, Son N. Tran, James C. Vickers, Quan Bai, Jane Alty

**Affiliations:** ^1^ Wicking Dementia Research and Education Centre University of Tasmania Hobart Tasmania Australia; ^2^ School of Psychological Sciences University of Tasmania Hobart Tasmania Australia; ^3^ Preventive Neurology Unit, Wolfson Institute of Population Health Queen Mary University of London London UK; ^4^ School of Allied Health, Human Services and Sport La Trobe University Melbourne Victoria Australia; ^5^ School of ICT University of Tasmania Hobart Tasmania Australia; ^6^ School of Information Technology Deakin University Melbourne Victoria Australia; ^7^ School of Medicine University of Tasmania Hobart Tasmania Australia; ^8^ Neurology Department Royal Hobart Hospital Hobart Tasmania Australia

**Keywords:** asymptomatic older adults, early Alzheimer's disease, episodic memory, hand motor function, keyboard tapping tests, motor‐cognitive, TAS Test

## Abstract

**INTRODUCTION:**

Finding low‐cost methods to detect early‐stage Alzheimer's disease (AD) is a research priority for neuroprotective drug development. Presymptomatic Alzheimer's is associated with gait impairment but hand motor tests, which are more accessible, have hardly been investigated. This study evaluated how home‐based Tasmanian (TAS) Test keyboard tapping tests predict episodic memory performance.

**METHODS:**

1169 community participants (65.8 ± 7.4 years old; 73% female) without cognitive symptoms completed online single‐key and alternate‐key tapping tests and episodic memory, working memory, and executive function cognitive tests.

**RESULTS:**

All single‐key (*R* ^2^_adj_ = 8.8%, ΔAIC = 5.2) and alternate‐key (*R* ^2^_adj_ = 9.1%, ΔAIC = 8.8) motor features predicted episodic memory performance relative to demographic and mood confounders only (*R* ^2^_adj_ = 8.1%). No tapping features improved estimation of working memory.

**DISCUSSION:**

Brief self‐administered online hand movement tests predict asymptomatic episodic memory impairment. This provides a potential low‐cost home‐based method for stratification of enriched cohorts.

**Highlights:**

- We devised two brief online keyboard tapping tests to assess hand motor function.
- 1169 cognitively asymptomatic adults completed motor‐ and cognitive tests online.
- Impaired hand motor function predicted reduced episodic memory performance.
- This brief self‐administered test may aid stratification of community cohorts.

## 1 BACKGROUND

Dementia prevalence is predicted to triple to more than 150 million globally by 2050.^1^, ^2^ Most cases are caused by Alzheimer's disease (AD), which has a 10‐ to 20‐year presymptomatic period of “silent” brain pathology (amyloid beta and tau deposition) before episodic memory symptoms emerge.^3^ To reduce dementia prevalence, there is an urgent need to identify at‐risk individuals for early interventions. Up to 40% of dementia cases are attributable to modifiable risk factors,^4^ and the recent emergence of monoclonal therapies for AD may be most efficacious earlier in the disease course.^5^, ^6^ Dementia prevention and drug development are hindered by the lack of accessible, cost‐effective tests to identify “at‐risk” cohorts for specialist assessments, early recruitment to clinical trials, and targeted interventions. Current methods, such as neuropsychological assessments, blood‐ and cerebrospinal fluid (CSF)‐based biomarkers, and positron emission tomography (PET) scans are too expensive, time‐consuming, or invasive.^3^

Motor analysis holds strong potential to identify people at risk of AD. Gait slows down, with less rhythmic stepping, about 10 years before dementia diagnosis,^7^ but the need for specialist movement‐sensing equipment and fall risk are barriers for translation to a population‐level test. Though less explored than gait, hand motor function also declines in presymptomatic AD and has the advantage of accessible assessments through computer keyboards.^8^ Two recent studies found slower and less rhythmic tapping features in mild cognitive impairment (MCI) and AD, but both required laboratory‐based equipment.^9^, ^10^ In 2019, Mollica et al. evaluated hand motor function in 72 older adults and found the speed and rhythm of repeatedly tapping a single computer key discriminated those with presymptomatic AD (*n* = 20, CSF Aβ‐positive) from those with negative CSF biomarkers (*n* = 37).^8^ These promising findings have not yet been replicated in other at‐risk groups or in larger samples.

This study builds upon Mollica et al.’s study by designing a self‐administered keyboard tapping test to measure hand motor function at home. We aimed to evaluate (i) whether a single‐key tapping test would help identify subtly impaired cognitive performance in episodic memory in a large community cohort of older cognitively asymptomatic adults, over and above a model that includes age, education, sex, level of education, anxiety, and depression; (ii) how a single‐key motor performance test would compare with a more challenging alternate‐key tapping test (based on the Bradykinesia Akinesia Incoordination [BRAIN] tap test^11^); and (iii) which combination of motor features would best predict cognitive performance.

We hypothesized that impaired hand motor performance (defined as slower tapping frequency, arrhythmia, inaccurate target, or prolonged dwell time on each key) would associate with impairments in episodic memory but not in cognitive domains less sensitive to early AD (working memory, executive dysfunction)^12^ and that the alternate‐key test would have a stronger association due to greater cognitive loads required for key switching.

## 2 METHODS

### 2.1 Study participants

Participants were recruited from a community sample of adults aged 50+ years residing in Tasmania, Australia who were enrolled in the Island Linking Aging and Neurodegenerative Disease (ISLAND) Project,^13^ which is a public health initiative that educates participants on how to modify their dementia risk factors. The ISLAND protocol has been described by Bartlett et al.^13^ Briefly, data are collected from participants via online self‐reported annual surveys, including age, sex, education (duration in years and highest level), depression and anxiety levels (via Hospital Anxiety and Depression Scale [HADS]), and information about diagnoses such as Parkinson's disease (PD), Multiple Sclerosis (MS), dementia, and heart disease. Between June and August 2021, ISLAND participants were invited via email to complete an online battery of motor‐cognitive tests, including keyboard tapping tests, via the TAS Test website.^14^ They completed online cognitive tests of Paired Associates Learning (PAL) and Spatial Working Memory (SWM) via the Cambridge Neuropsychological Test Automated Battery (CANTAB) in August 2021.

**RESEARCH IN CONTEXT**

1. **Systematic review**: We reviewed journal articles and abstracts using PubMed and Google Scholar. Emerging evidence suggests motor analysis holds strong potential to identify people at risk of AD. Relevant citations are cited.
2. **Interpretation**: 1169 cognitively asymptomatic adults aged 50 to 90 competed self‐administered Tasmanian (TAS) Test keyboard tapping tasks. All motor features of single‐key and alternate‐key tapping tests improved estimation of episodic memory performance relative to models with demographic and mood confounders only.
3. **Future directions**: Brief self‐administered online hand motor tests may aid identification of presymptomatic episodic memory decline. This provides a potential low‐cost, accessible method for stratification of enriched cohorts for further assessment. Future work should compare the keyboard tapping tests to other biomarkers of presymptomatic AD, such as blood‐based biomarkers and PET scans. Prospective longitudinal designs are required to support the association between motor performance and cognitive function.

As we aimed to detect subtle cognitive deficits in people without cognitive symptoms, those who answered yes to any of the following questions were excluded from analysis: 1. “Have you noticed a substantial change in your memory and mental function in recent years?” 2. “Have you been told by a doctor that you have dementia?” 3. “Have you been told by your doctor that you have memory impairment but you are uncertain if you have dementia?” Participants with a self‐reported PD or MS diagnosis were also excluded. Information about cognitive symptoms, PD, and MS diagnosis were obtained through an online questionnaire.

The completion rate of CANTAB in our cohort was about 70%; the 30% who did not complete the cognitive tests were removed from the sample prior to the stated sample size. Participants who completed the online cognitive tests but did not complete the keyboard tapping tests were excluded from the analysis. We also compared the cognitive scores (ie, PALTEA6, SWMBE6, SWMS, respectively) between the group that completed the online cognitive tests and the keyboard tapping tests with the group that completed just the online cognitive test.

### 2.2 Ethics and consent

The Human Research Ethics Committee of the University of Tasmania approved the TAS Test Project (ref. HREC H0021660; registered on the ClinicalTrials.gov registry as NCT05194787) and the ISLAND Project (ref. HREC H001864). All participants gave informed consent.

### 2.3 Keyboard tapping tasks

**FIGURE 1:**
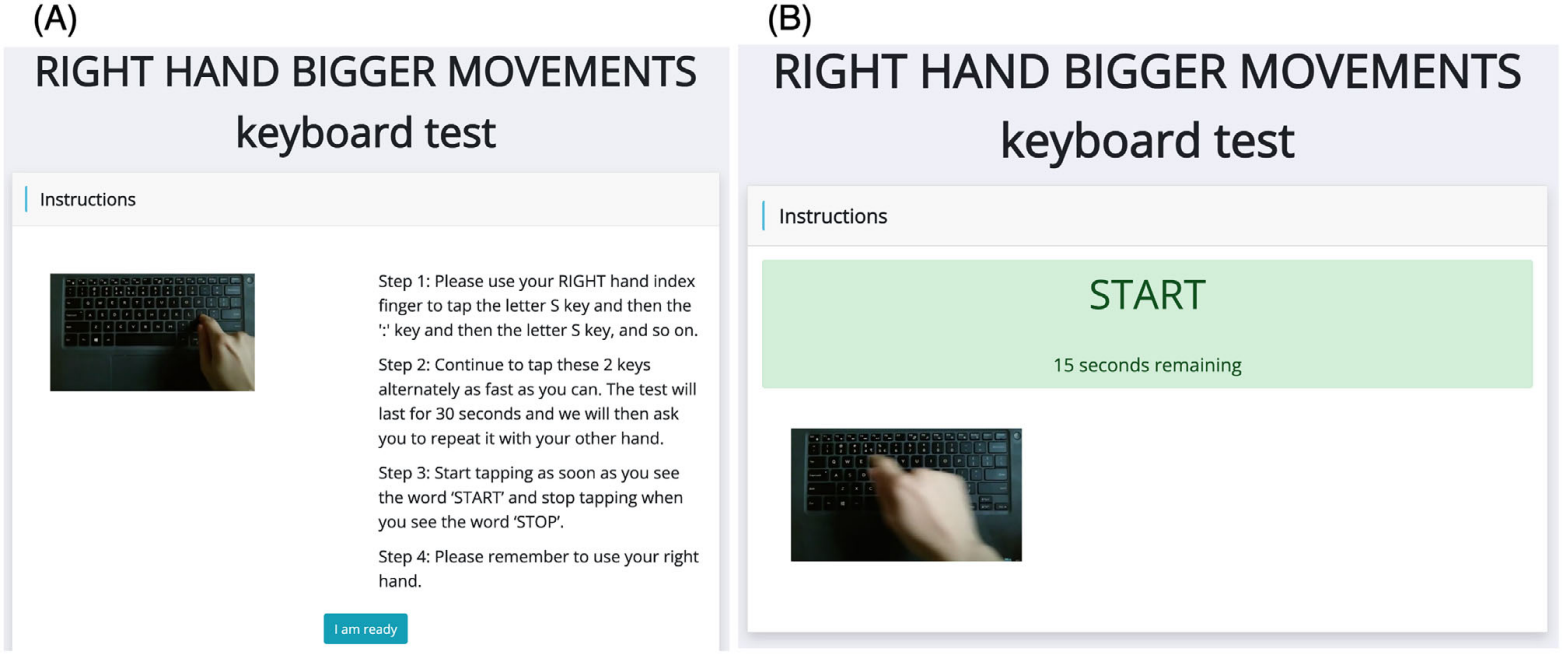
Screen shots of alternate‐key tapping test from TAS Test website: (A) instructions screen and (B) recording screen. The looped video of the hand tapping the correct keys plays automatically on both the instruction and the recording screen to remind the participants what to do throughout the test. The recording screen was shown after the participant pressed the blue “I am ready” button on the bottom of the instruction screen. There was a 10 s countdown on the recording screen before the green “START” bar was presented. A numerical countdown showed how many seconds of recording remained, and this was replaced by a red “STOP” bar (with a numerical countdown but no looped video) during the 30 s rest periods between blocks of tapping.

The keyboard tapping tests were performed as part of the TAS Test protocol, previously described by Alty et al.^14^ Briefly, this self‐administered online battery of tests aims to detect preclinical AD and prospectively predict cognitive decline using standard home desktop and laptop computers. Self‐reported hand dominance was recorded. Participants followed on‐screen test instructions^14^ (Figure 1A). In the TAS Test protocol, the single‐key tapping test is similar to the test used in Mollica et al.’s study and the alternate‐key tapping test closely replicates the BRAIN tap test.^15^ The BRAIN test was first developed in 1999 and subsequently revised as a computerized assessment of upper limb motor function in PD; it has been validated against clinical rating scales in PD and MS^11^, ^16^, ^17^ but had not previously been evaluated alongside tests of cognitive performance. The order of the tapping tests was fixed, with the single‐key test first.

#### 2.3.1 Single‐key tapping test

Participants tapped the spacebar with their dominant hand as quickly as possible for 10 s, repeated for four blocks in total, with a 30 s break between each block. The TAS Test protocol differs slightly from Mollica et al.’s method of six blocks of 10 s tapping periods on the spacebar, alternating between each hand with a 30 s break between blocks.^8^ Because there was no difference between either hand's performance in Mollica et al.’s study and we were focusing on making a brief, simple online test, we shortened the test duration.

#### 2.3.2 Alternate‐key tapping test

The TAS Test alternate‐key tapping test was devised in consultation with the developers of the BRAIN Tap test (author AN) to tap the same keys but to reduce the duration of tapping from 3 to 1 min, without any practice trials, for the purpose of having a shorter test that could be delivered online in remote settings. Participants tapped the “S” and “;” keys alternately with their right hand as fast as possible for 30 s and then repeated this with their left hand; see Figure 1B. In the original BRAIN tap test, participants had two 30 s practice tests before four 30 s tapping tests, alternating tests between each hand.

### 2.4 Cognitive performance

Participants completed two online cognitive assessments, PAL and SWM, from the CANTAB battery, validated in MCI and AD.^18^, ^19^, ^20^, ^21^, ^22^

#### 2.4.1 Episodic memory assessment

**FIGURE 2:**
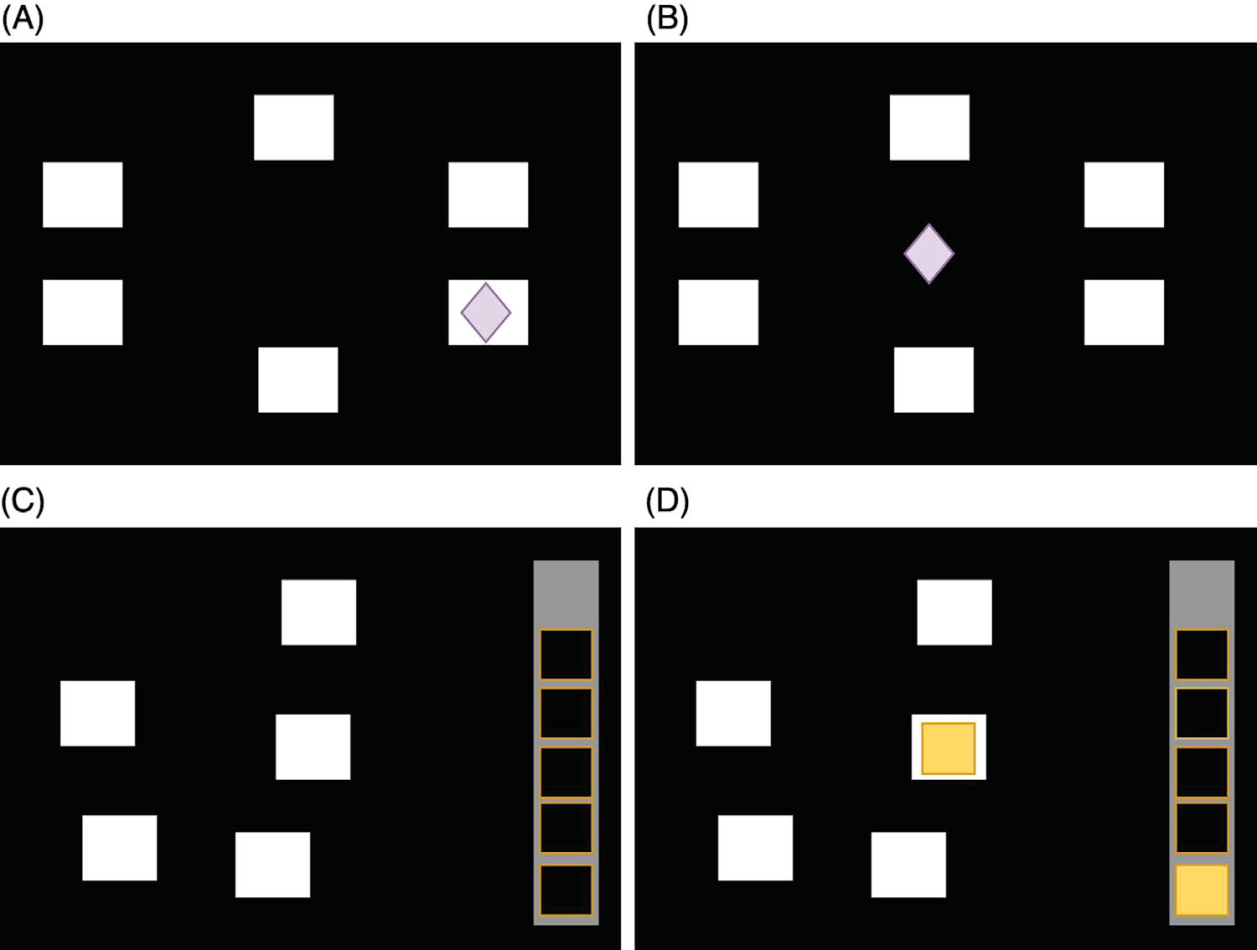
Schematic diagram of stages of PAL and SWM cognitive tests. PAL: (A) Participants were asked to remember how different patterns were paired with their original locations (ie, which pattern was associated with which box) on the computer screen. Six boxes were arranged in a circle, and boxes were “opened” one by one randomly by the computer program to reveal a pattern in just one box. (B) After all boxes had been opened, the pattern (diamond) was shown in the center of the screen, and participants were required to click the box in which the pattern was previously located. SWM: (C) Boxes were presented and participants must click on each box to find the location of a token (yellow square). They were provided with a key piece of information—that yellow tokens would only appear once in each box per trial, that is, the tokens would never appear twice in one box. (D) When a token was found, it was added to the vertical gray bar, and participants then had to click on the boxes again to find the next token. In each trial, the total number of boxes increased, and the number of boxes containing a hidden token also increased from three to four and then to six boxes.

The PAL assesses visual associative and episodic memory, and typical administration time is 8 min; see Figure 2. The number of hidden patterns increases from one in the first stage to eight in the last stage. The PALTEA6 score is the total errors at the six‐pattern stage, adjusted for incomplete or failed trials. PAL scores distinguish between older adults with MCI and healthy controls with a sensitivity/specificity of 0.83/0.82,^18^ and longitudinal decline predicts progression from MCI to AD.^19^

#### 2.4.2 Working memory and executive function assessment

The SWM test requires participants to search for hidden yellow tokens by clicking on boxes presented on the screen to “open” them; see Figure 2. The number of times the participant revisits a box in which a token had previously been found is the “between errors score” and measures spatial working memory; the between errors adjusted for a six‐token stage is the SWMBE6 score. The strategy score (SWMS) is a measure of executive function (planning) and defined as the number of distinct boxes used by the participant to begin a new search for a token, that is, choosing the same box to start with each time and following a predetermined searching sequence indicates good planning abilities. Sahgal et al. found worse SWM performance in AD compared to healthy controls.^21^

### 2.5 Data analysis

**FIGURE 3:**
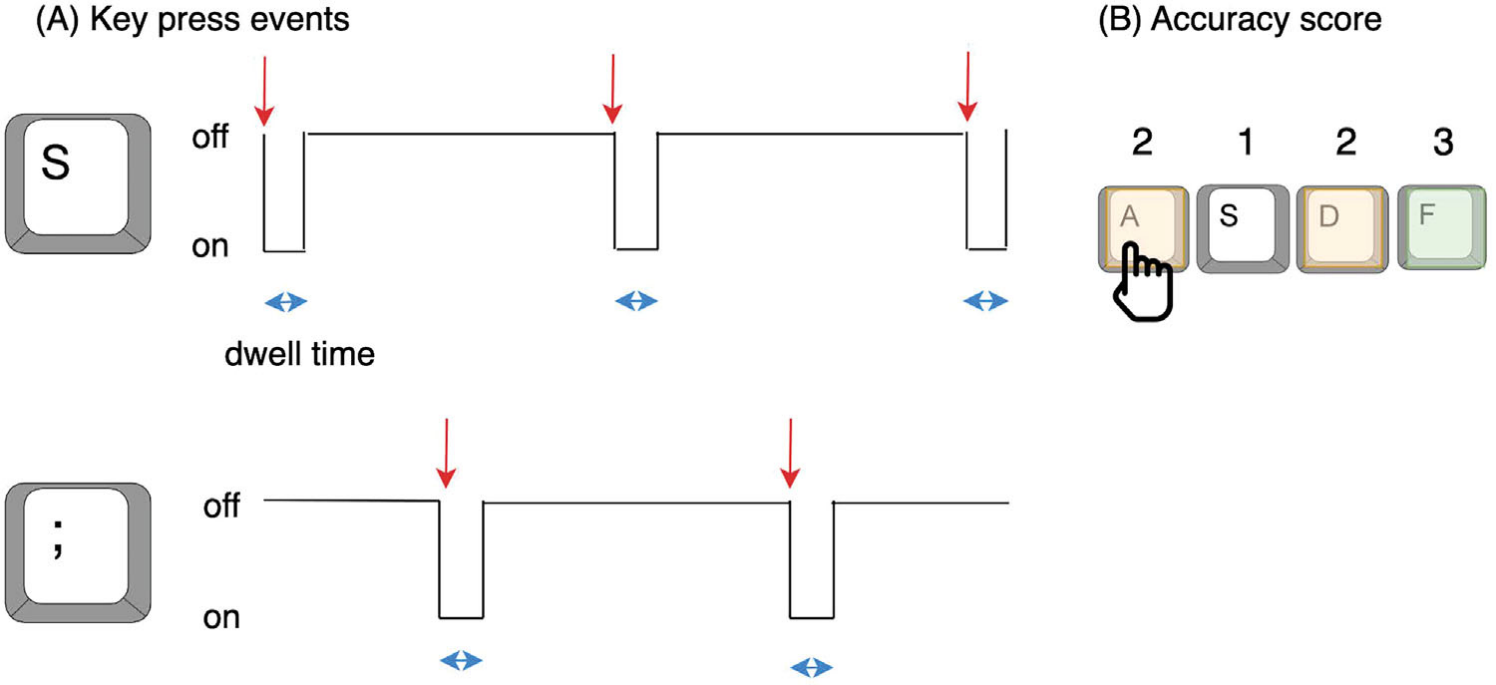
Schematic representation of key press events in the TAS Test alternate‐key tapping test from which motor features were calculated. The same features were also extracted for the TAS Test single‐key tapping test. (A) Red arrows indicate when a key was fully pressed (on), which is used to calculate frequency (number of key presses per 1 s period) and variability (standard deviation of log time interval between key presses). Blue arrows indicate dwell time, which is the duration, in milliseconds, that keys are pressed (on). (B) The accuracy score is a weighted index using the number of keys incorrectly pressed scored in a target fashion (1 point for the correct key, 2 points for adjacent keys, and 3 for other keys) divided by the total number of key presses, that is, if all keys are pressed correctly, the accuracy score is 1.0.

Each key‐press event was recorded in milliseconds, and measures of motor function were calculated based on formulas from previous BRAIN tap papers: tapping frequency, variability (rhythm), dwell time, and accuracy (Figure 3).^23^ To test whether keyboard tapping features improved prediction of cognitive performance over and above demographic and mood scores, we compared models with and without the motor features included.

By definition, keyboard tapping features are dependent, since they are transformations of the same data, so we compared models using Akaike's information criterion (AIC) rather than variable selection using *p* values. We used an automated procedure (the “dredge” function from the MuMIn R package^24^, ^25^) to fit models with every linear, additive combination of motor features (not allowing for interactions), fixing the covariates age (in years), sex (male/female), level of education (categorical), depression scores, and anxiety scores (from HADS) so they were included in each model. For each cognitive measurement (eg, PALTEA6, SWMBE6, SWMS), three discrete sets of motor features were considered: (i) all single‐key tapping features, including features from all four blocks; (ii) all alternate‐key tapping features, including features from both the dominant and non‐dominant trials; and (iii) all single‐key and alternate‐key tapping features combined. We included all participants in all the regression analyses conducted. For each set, and each dependent variable, a single hypothesis was considered, whether any keyboard tapping features were included in the ΔAIC > 2 set. In total, nine hypotheses were tested. Models were ordered by AIC (with finite sample correction). Models with ΔAICij < 2 were considered statistically equivalent, where ΔAICij is the difference between AIC for model *i* and model *j* with the lowest AIC.^26^ If the set of statistically equivalent models (ΔAIC < 2) did not include the model containing only covariates and no keyboard tapping features (which we refer to as the “null model”), then we could conclude that keyboard tapping features improved prediction of cognitive test scores over and above the included covariates. Penalized likelihood approaches tend to select parsimonious models with good out‐of‐sample predictive performance.

Cognitive test scores were counts, so we fitted generalized linear models assuming residuals were Poisson or negative binomial distributed. The negative binomial models were better fits according to ΔAIC > 2. However, for SWM between errors the covariate‐only model would not converge, so we assumed residuals were normally distributed.

## 3 RESULTS

CANTAB was completed by 2766 participants. There were 1407 participants who completed the online cognitive tests but did not complete the keyboard tapping tests, and these participants were excluded from the analysis. The most common reason stated was that their only computer device at home was a tablet (eg, iPad) or smartphone (ie, lack of a computer keyboard). There was no significant difference in mean cognitive scores between the group who completed both the online cognitive tests and the keyboard tapping tests (*n* = 1359) and the group that completed the online cognitive tests only (*n* = 1407): PALTEA6 (means = 4.49 and 4.69 for the former and latter groups, respectively, *p* = .29), SWMBE6 (means = 3.32 and 3.31 for the former and latter groups, respectively, *p* = .94), and SWMS (means = 7.82 and 7.97 for the former and latter groups, respectively, *p* = .16). Participants (*n* = 105) who had not finished all single‐key (four blocks) and all alternate‐key tapping tests (dominant hand and non‐dominant hand) were also excluded from analysis. 1254 participants completed the keyboard and cognitive tests, but 85 were excluded from the analysis due to cognitive symptoms (*n* = 74) or diagnosis declared in the medical surveys (PD [*n* = 3], MS [*n* = 8]). Table 1 summarizes the demographic and cognitive data from the remaining sample of 1169 cognitively asymptomatic participants who were included in the analysis. Descriptive statistics are presented in Table 2, and correlation matrix of tapping features are shown in Figure A1.

**TABLE 1:** Demographics of community cohort of cognitively asymptomatic adults.

	Overall (*N* = 1169)
Age (years)	
Mean (SD)	65.8 (7.37)
Median [Min, Max]	66.0 [51.0, 89.0]
Sex	
Female	854 (73.1%)
Male	315 (26.9%)
Educational attainment	
Left formal education before 16 years old	49 (4.2%)
Left formal education at age 16	108 (9.2%)
Left formal education at age 17 or 18	162 (13.9%)
Undergraduate degree or equivalent	577 (49.4%)
Master's degree or equivalent	202 (17.3%)
PhD or equivalent	71 (6.1%)
Retired	
Yes	769 (65.8%)
No	389 (33.3%)
Not disclosed	11 (0.9%)
HADS Anxiety	
Mean (SD)	4.28 (3.55)
Median [Min, Max]	3.00 [0, 19.0]
HADS depression	
Mean (SD)	2.90 (2.72)
Median [Min, Max]	2.00 [0, 15.0]
PAL (total errors 6‐pattern adjusted)	
Mean (SD)	4.40 (4.80)
Median [Min, Max]	3.00 [0, 22.0]
SWM (between errors 6‐pattern)	
Mean (SD)	3.22 (3.32)
Median [Min, Max]	2.00 [0, 13.0]
SWM (strategy)	
Mean (SD)	7.75 (2.76)
Median [Min, Max]	8.00 [2.00, 13.0]

Abbreviations: HADS, Hospital Anxiety and Depression Scale; Max, maximum; Min, minimum; PAL, paired associates learning; SD, standard deviation; SWM, spatial working memory.

**TABLE 2:** Descriptive statistics of TAS Test keyboard tapping features.

	Overall (*N* = 1169)
Single‐key tapping frequency (Key presses/second)	
Block 1	
Mean (SD)	5.54 (0.813)
Median [Min, Max]	5.62 [2.12, 8.37]
Block 2	
Mean (SD)	5.62 (0.839)
Median [Min, Max]	5.68 [1.73, 8.70]
Block 3	
Mean (SD)	5.70 (0.860)
Median [Min, Max]	5.71 [1.45, 8.77]
Block 4	
Mean (SD)	5.77 (0.871)
Median [Min, Max]	5.81 [1.43, 8.70]
Alternate‐key tapping frequency (Key presses/second)	
Dominant hand	
Mean (SD)	2.17 (0.365)
Median [Min, Max]	2.16 [1.12, 4.65]
Non‐dominant hand	
Mean (SD)	2.00 (0.321)
Median [Min, Max]	2.00 [1.06, 3.12]

Abbreviations: Max, maximum; Min, minimum; SD, standard deviation; sec, second. Each block of single‐key tapping (with the dominant hand) was 10 s long with a 30 s break between blocks. Each alternate‐key tapping period for the dominant and non‐dominant hands was 30 s with a 30 s break in between.

### 3.1 Keyboard tapping prediction of episodic memory performance

The null model, comprising covariates of age, sex, level of education, depression scores, and anxiety scores, had an AIC = 5969.7 (*R* ^2^_adj_ = 8.1%) for predicting episodic memory performance (PALTEA6). When motor features from the single‐key tapping test were included, the highest ranked model's estimation of episodic memory improved to AIC = 5964.5 (*R* ^2^_adj_ = 8.8%) with a ΔAIC = 5.2 (where ΔAIC > 2 denotes statistical significance). All motor features from the single‐key tapping tests were equivalent in terms of improving the prediction of episodic memory performance (ΔAIC < 2) except for trial 3 variability and trial 4 accuracy scores (which were slightly inferior). For the alternate‐key tapping test, the motor features improved the prediction of episodic memory performance; the highest ranked model had an AIC = 5960.8 with an *R* ^2^_adj_ = 9.1% and ΔAIC 8.8, compared to the null model. All alternate‐key keyboard tapping features were equally good at predicting episodic memory performance (ΔAIC < 2).

When comparing the two tapping tests, the alternate‐key motor features (AIC = 5960.8, *R* ^2^_adj_ = 9.1%) were better than the single‐key features (AIC = 5964.5, R^2^_adj_ = 8.8%) at estimating episodic memory (ΔAIC > 2). The coefficients and incident rate ratios (>1 indicates a higher rate of errors and <1 indicates a lower rate) for all models comprising motor features of the alternate‐key tapping test that substantially improved the prediction of episodic memory compared to the null model are presented in Table B1. Combining the single‐key and alternate‐key tapping features was no better than the alternate key tapping test on its own (ΔAIC < 2). In summary, motor features from the keyboard tapping tests, regardless of the specific protocol or duration, improved prediction of episodic memory over and above the null model.

**FIGURE 4:**
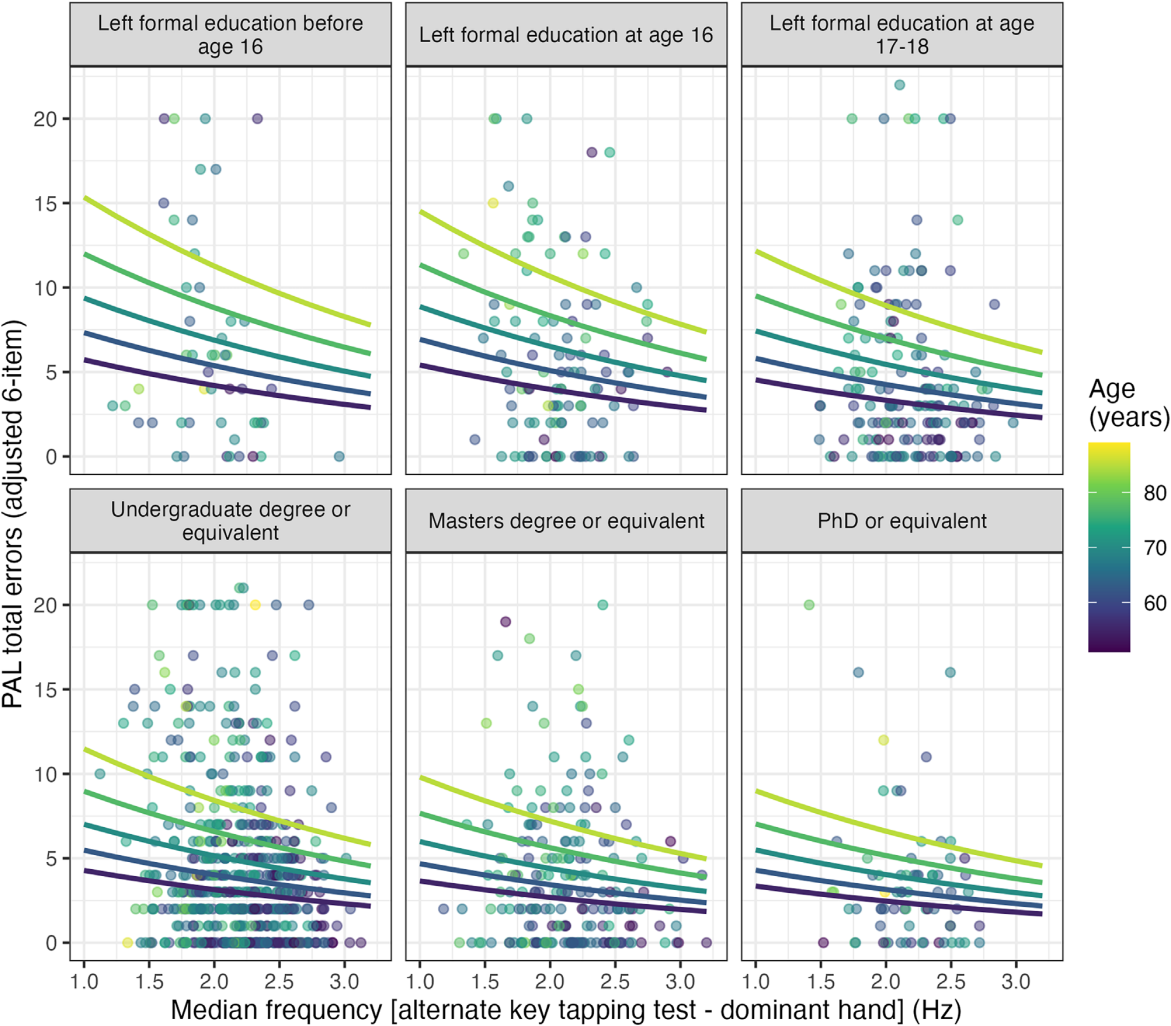
Episodic memory by dominant hand alternate‐key tapping frequency compared across age and level of education. The trend curves show expected episodic memory performance (PAL total errors adjusted for six‐pattern stage) with more errors associated with low tapping frequencies and older age. Non‐dominant hand tapping variability, anxiety and depression scores, and sex are fixed at their respective means. This model predicts PAL total error scores significantly better than the covariate‐only null model (ΔAIC = 8.8). The findings also demonstrated that while episodic memory performance was reduced in older participants, this was of a greater magnitude in individuals with lower educational attainment, possible indicative of the role of cognitive reserve.

The highest ranked model for predicting episodic memory used coefficients of dominant hand tapping frequency and non‐dominant hand variability (AIC = 5960.8; *R* ^2^_adj_ = 9.1%; ΔAIC = 8.8 compared to null model). Figure 4 presents the expected PAL total error scores conditioned on dominant hand tapping frequency, age, and level of education predicted by this model (other covariates and non‐dominant hand tapping variability held fixed at their respective means^27^).

### 3.2 Keyboard tapping prediction of executive function

The null model's AICs were 5297.6 for SWM between errors, six tokens (BE6), and 5521.9 for SWM strategy, with *R* ^2^_adj_ = 5.8% and 15%, respectively. The addition of motor features from the single‐key test did not improve estimation of SWM measured by BE6 but did improve the estimation of strategy (ΔAIC = 5.8, AIC = 5516.1, *R* ^2^_adj_ = 15.8%). The addition of motor features from the alternate‐key test was no better than the null model at predicting SWM, with each ΔAIC < 2. Thus, in contrast to the episodic memory predictions, the addition of motor tapping features did not improve the prediction of working memory, and only the single‐key tapping test improved the estimation of strategy.

## 4 DISCUSSION

In a large community cohort of 1169 older adults that only included those without any cognitive symptoms or diagnoses, we found that brief, self‐administered tapping tests were able to identify subtle reductions in performance in the episodic memory cognitive domain relative to peers of the same age, sex, education, and mood. The single key (40 s) and alternate key (60 s) tapping tests both demonstrated associations with episodic memory, but neither improved the identification of performance in the working memory domain. The results suggest movement characteristics of repetitive keyboard tapping reflect episodic memory function.

Studies have shown that episodic memory deficits in cognitively asymptomatic older adults are associated with increased risk for AD.^28^, ^29^, ^30^ Consistent with these findings, it has been found that the pathological alterations of AD occur in the hippocampus, and other neighboring regions that are critical to episodic memory function, long before an AD diagnosis is made.^31^ It is unclear why a repetitive motor task was more associated with episodic memory than executive function, which involves motor planning. The task may be sensitive to degradation in attentional processes needed to encode episodic memories.^32^ As episodic memory is the first cognitive domain to decline in most people with AD,^30^ and the cohort comprised only those without cognitive symptoms, these results point to the potential of simple keyboard tapping tests to form part of a population‐level strategy to stratify risk of presymptomatic AD.

To the best of our knowledge, only one previous study examined single‐key tapping in precognitive AD, and this was in a small sample (*n* = 72) in a research setting; the authors found a significant association between single‐key tapping speed and variability with cerebrospinal fluid Aβ levels (“preclinical AD”) in participants with normal cognition.^8^ Our results align with this finding as speed and variability of all four single‐key tapping blocks, except the tapping variability of the third block, were significant predictors of episodic memory performance. Variability was also included in the highest ranked predictive model for single‐key tapping (ΔAIC = 5.2). The present study expanded upon this work: first, we replicated the study in a larger cohort (*n* = 1169 vs. 72), used a self‐administered home‐based test rather than a research setting, and reduced test durations to 40 s. We also expanded the motor measures beyond speed and variability and found dwell time and inaccuracy score further improved the prediction of episodic memory.

The vast majority of previous keyboard tapping studies primarily used keyboard tapping to measure motor decline directly rather than to assess how hand motor function helped predict decline in cognitive function. They were mostly undertaken in PD with only a few in AD dementia and MCI—and none in large non‐symptomatic community samples; a recent meta‐analysis of 41 keyboard tapping studies included 254 participants with MCI and AD; the authors found keystroke features had a pooled sensitivity/specificity of 0.85/0.82 for discriminating symptomatic cognitive impairment from healthy controls.^33^ However, all reviewed studies used equipment (eg, pressure transducers or mobile phone applications) rather than standard computer keyboards to collect tapping features. The TAS Test project, comprising the single and alternate keyboard tapping tests, is unique in that it focuses on using motor tests to detect presymptomatic declines in cognitive function and other biomarkers of AD pathology; furthermore, all tests are designed to be self‐administered at home rather than researcher‐led.^14^

We hypothesized that the alternate‐key tapping test would have stronger associations with episodic memory than the single‐key test as it is a more challenging test, and our results supported that hypothesis. In addition, tapping variability was in the best performing models for both the single‐key and alternate‐key tapping tests. The fact that both models had the same motor features of variability suggests the ability to maintain a consistent motor rhythm is associated with episodic memory function. The results for executive function were mixed, with only the single‐key test associating and executive function. Further study of different cohorts will be required to clarify this. The findings from the present study on motor‐cognitive associations are supported by the established evidence base around gait analysis in preclinical AD and the motoric‐cognitive risk syndrome^34^; although the underlying pathology for these associations remains uncertain, several studies found associations between episodic memory with longer stride time (reduced step frequency) and greater variability of stride time.^35^, ^36^, ^37^

The strengths and limitations of the study are acknowledged. Its strengths include the large community cohort with a wide range of ages and cognitive scores, the use of validated online cognitive tests, measurement of different cognitive domains, and careful adjustment for covariates that are known to associate with both cognitive and motor test performance, including depression and anxiety. Furthermore, using a self‐administered online protocol obviated the need for researcher/clinician involvement, increased accessibility, and minimized geographical or cost barriers. CANTAB PAL and SWM generally take approximately 12 min to complete, and the completion rate in our cohort was about 70%. In our asymptomatic group, we use CANTAB to detect the most subtle areas of decline and have shown that at this very early stage motor function also declines, so the two together could be supportive in identifying people at risk of AD. Keyboard tapping tests can be done much quicker and more easily compared to CANTAB. Further, keyboard tapping tests are free to use at no cost, whereas CANTAB charges for both PAL and SWM. The study limitations include the cross‐sectional design, so it remains unclear whether declines over time in episodic memory are associated with keyboard tapping. Also, although the sample was community based, it was recruited from a cohort of participants enrolled in a dementia prevention study and so is biased toward those who are healthy and educated (more than 50% have attained university‐level education or above). It is also predominantly a female cohort (72%). The ISLAND cohort recruited participants from Tasmania, and according to the Australian Bureau of Statistics 2021 census, most of the older adults in Tasmania are White and of Northern European ancestry.^38^ Thus, representativeness and generalizability to the broader population are limited. We adjusted for several confounding variables known to affect tapping performance: depression, anxiety, education, age, and sex. We excluded people with a diagnosis of PD or MS. However, additional variables that could have impacted performance, such as arthritis, joint pain, other neurological and psychiatric disorders, and visual function, were not accounted for as they were not specifically addressed in the online questionnaire. Time of day and stimulant or medications that could positively or negatively impact tapping performance were also not measured as part of the online test. Future work will include these variables in our analysis. It is recognized that the hand dominance was determined by a single question rather than a validated handedness questionnaire. As the test was conducted in a home environment with no researcher assistance, it is not guaranteed that participants completed the test correctly, eg, some participants may have used two hands to tap alternate keys rather than one. Furthermore, variations between home computer keyboards and software may have introduced delays in key‐press recordings. However, using an asymptotically large sample, AIC performs well in selecting models that would perform well at predicting episodic memory out of sample (using data that the model was not trained on). Model selection and comparison are less affected by influential observations and outliers than model selection using *p* values (eg, stepwise regression procedures), so for these reasons we expect our findings to be robust to errors caused by chance differences in home computers, falsely recorded exceptional or poor performance, or difficulty following instructions.

In summary, through a large community cohort of adults without any cognitive symptoms or diagnoses, we found an association between the TAS Test single‐key and alternate‐key tapping tests with episodic memory performance, but there was no consistent association between the tapping tests and executive function. Both hand motor tests predicted episodic memory performance over and above the null model (comprising demographic and mood data), with the alternate‐key test predicting better than the single‐key test. Our study demonstrated an association between keyboard tapping and cognitive performance. We recognize the limitation of a cross‐sectional study and that we cannot be certain that people with lower cognitive performance have presymptomatic AD because participants were asymptomatic without any AD diagnosis at the time of data collection. Prospective longitudinal studies are required to further explore the feasibility of using tapping features to risk stratify presymptomatic AD. We plan to address this limitation by following up with participants over time and inviting them to complete CANTAB every 2 years. Future work should also include comparison of the keyboard tapping tests to other validated biomarkers of presymptomatic AD risk, such as blood‐based biomarkers and PET scans. The association between cognitive function and tapping features in working versus retired individuals should also be analyzed in the future.

## CONFLICTS OF INTEREST STATEMENT

The authors declare no conflicts of interest.

## CONSENT STATEMENT

All human subjects provided informed consent.

## Supporting information

Supporting Information

Supporting Information

Supporting Information
