# Supplementary material for: Estimating presymptomatic episodic memory impairment using simple hand movement tests: A cross‐sectional study of a large sample of older adults
Source: Alzheimers Dement. 2023 Jul 30;20(1):173–82. doi: 10.1002/alz.13401 (PMC10916999; doi:10.1002/alz.13401)
Supplement: Supplementary file 2 — Supporting Information [file ALZ-20-173-s002.docx]

**Supplementary A**

**
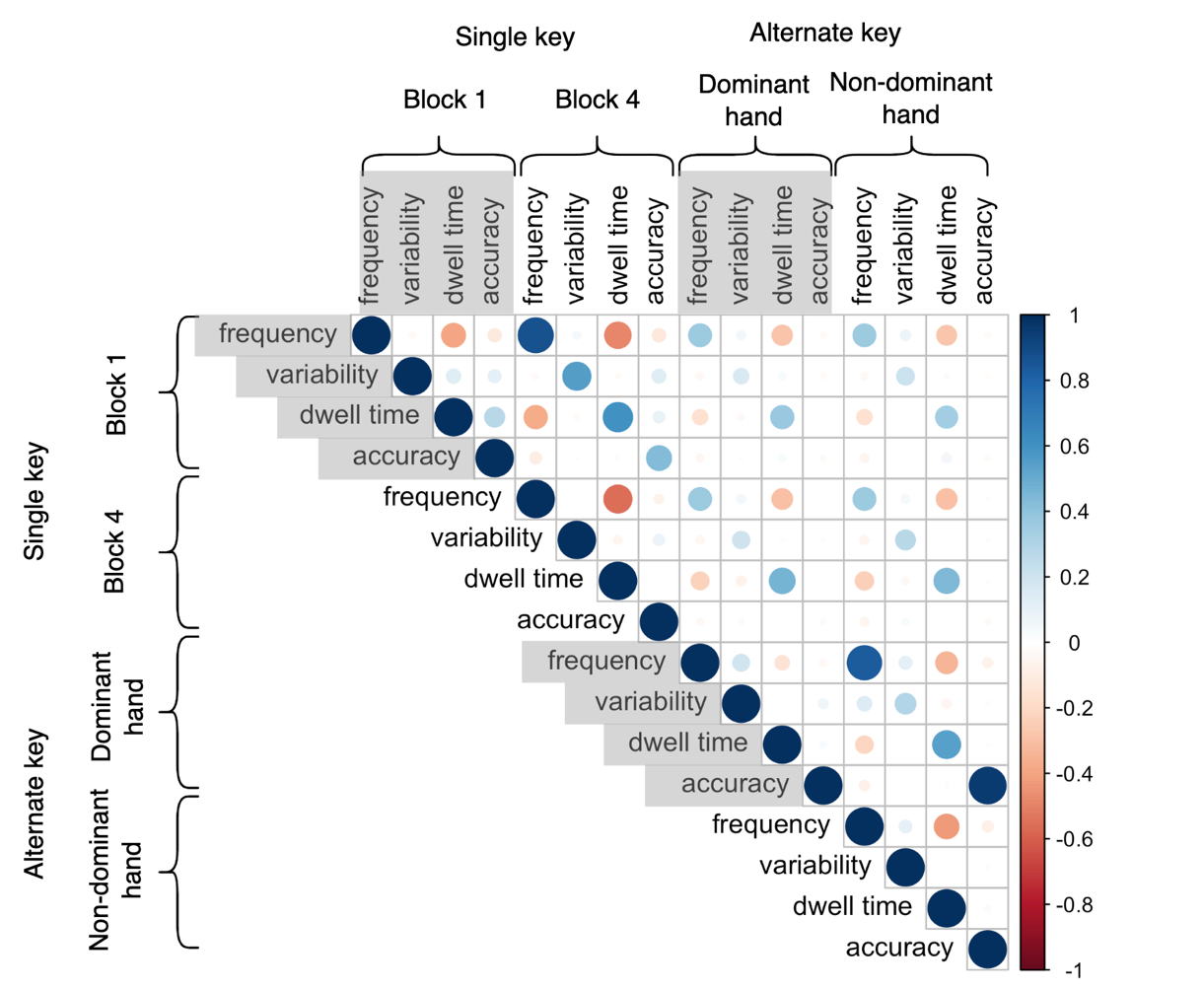
**

**Figure 1.** Correlation matrix of keyboard tapping features extracted from the first and the fourth blocks of the single key tapping tests and the dominant and non-dominant hand alternate key tapping tests. The size of the circles denotes the strength of linear relationships between tapping features. The intensity of the shading color denotes strength of correlations with red shades representing negative correlations and blue shades representing positive correlations.
