# Supplementary material for: Estimating presymptomatic episodic memory impairment using simple hand movement tests: A cross‐sectional study of a large sample of older adults
Source: Alzheimers Dement. 2023 Jul 30;20(1):173–82. doi: 10.1002/alz.13401 (PMC10916999; doi:10.1002/alz.13401)
Supplement: Supplementary file 3 — Supporting Information [file ALZ-20-173-s001.docx]

**Supplementary B**

**Table 1: Comparison of how various combinations of motor features predict episodic memory.** The models are ranked in order, from lowest to highest left to right, determined by AIC. Only models with ΔAIC>2 compared to null model are included. Episodic memory is measured by PAL total errors adjusted for 6-pattern stage * all confounders were removed from the table as they were all fixed in all models. Incidence Rate Ratios were reported for all variables included in the models.

| **Dwell time (dominant)** | - | - | - | - | - | - | - | - | 0.00 |
| --- | --- | --- | --- | --- | --- | --- | --- | --- | --- |
| **Incidence Rate Ratios** | - | - | - | - | - | - | - | - | 1.00 |
| **Dwell time (non-dominant)** | - | - | - | - | - | - | - | 0.00 | - |
| **Incidence Rate Ratios** | - | - | - | - | - | - | - | 1.00 | - |
| **Inaccuracy score (dominant)** | - | - | -0.10 | - | - | - | -0.10 | - | - |
| **Incidence Rate Ratios** | - | - | 0.91 | - | - | - | 0.90 | - | - |
| **Inaccuracy score (non-dominant)** | - | -0.10 | - | - | - | -0.10 | - | - | - |
| **Incidence Rate Ratios** | - | 0.90 | - | - | - | 0.90 | - | - | - |
| **Frequency (dominant)** | -0.31 | -0.32 | -0.31 | -0.33 | -0.25 | -0.34 | -0.34 | -0.32 | -0.31 |
| **Incidence Rate Ratios** | 0.73 | 0.73 | 0.73 | 0.72 | 0.78 | 0.71 | 0.71 | 0.73 | 0.74 |
| **Frequency (non-dominant)** | - | - | - | - | -0.08 | - | - | - | - |
| **Incidence Rate Ratios** | - | - | - | - | 0.92 | - | - | - | - |
| **Variability (dominant)** | - | - | - | 0.12 | - | 0.12 | 0.13 | - | - |
| **Incidence Rate Ratios** | - | - | - | 1.13 | - | 1.13 | 1.14 | - | - |
| **Variability (non-dominant)** | 0.25 | 0.25 | 0.25 | 0.22 | 0.25 | 0.22 | 0.21 | 0.25 | 0.25 |
| **Incidence Rate Ratios** | 1.28 | 1.28 | 1.28 | 1.24 | 1.29 | 1.24 | 1.23 | 1.28 | 1.28 |
| **RMSE** | 4.54 | 4.53 | 4.54 | 4.53 | 4.54 | 4.53 | 4.53 | 4.54 | 4.54 |
| **adjusted R^2** | 0.09 | 0.09 | 0.09 | 0.09 | 0.09 | 0.09 | 0.09 | 0.09 | 0.09 |
| **AICc** | 5960.82 | 5961.66 | 5961.79 | 5961.85 | 5962.66 | 5962.66 | 5962.67 | 5962.76 | 5962.77 |
| **ΔAICc** | 0.00 | 0.83 | 0.97 | 1.03 | 1.84 | 1.84 | 1.85 | 1.94 | 1.94 |

RMSE, root-mean-square deviation

-Indicates that variable is not included in the model, coefficients are on log-odds scale.
